# Treating critically ill patients with probiotics: Beneficial or dangerous?

**DOI:** 10.1186/1757-4749-3-2

**Published:** 2011-02-27

**Authors:** Christoph A Jacobi, Christian Schulz, Peter Malfertheiner

**Affiliations:** 1 Universitätsklinikum Magdeburg, Klinik für Gastroenterologie, Hepatologie und Infektiologie, Leipzigerstr. 44, 39120 Magdeburg, Germany

## Abstract

Probiotic bacteria are live microorganisms which confer to health benefits of the host. They help to maintain the integrity of the intestinal barrier function by modulating the mucosal and systemic immune response of the host. These bacteria have proven their beneficial effect in several conditions of ulcerative colitis. More recently probiotics/synbiotics have been included in the treatment of critically ill patients. However to date it remains uncertain whether probiotics/synbiotics are beneficial or even dangerous to the clinical outcome of this patient group. This article reviews the current evidence of the use of bacteria in critically ill patients in intensive care settings.

## Introduction

Severe sepsis with associated multisystem organ dysfunction is a leading cause of death in patients hospitalized in intensive care units (ICU). The gastrointestinal tract plays an important role in the pathogenesis of multiorgan dysfunction owing to a breakdown of intestinal barrier function and increased translocation of bacteria and bacterial components into the systemic circulation. During critical illness, alterations in gut microflora are due to several factors that include changes in circulating stress hormones, gut ischemia, immunosuppression, the use of antibiotics and other drugs, possible bacterial translocation and the lack of nutrients [1]. In experimental models of pancreatitis, it has been demonstrated, that *Lactobacillus *strains disappear after 6 to 8 hours from the gut and are replaced by an overgrowth of pathogenic bacteria [2]. The importance of endogenous strains of probiotic bacteria such as *Bifidobacterium *and *Lactobacillus *in maintaining intestinal barrier function by modulating mucosal and systemic immune responses is becoming evident from numerous studies [3]. Also the airways can be colonized by pathogens, due to broad-spectrum antibiotic use, reduction in gastric pH as part of the stress ulcer prophylaxis, and the impairment of mucosal defense mechanisms due to trauma induced by indwelling endotracheal or nasogastric tubes [4]. In fact, ventilator associated pneumonia (VAP) occur in 9-27% of patients maintained on mechanical ventilation for more than 48 h. VAP is associated with a mortality of 10-40%, increased ICU and hospital stay and an estimated cost of between US$ 12.000 to US$ 16.000 per episode [5,6]. Thus, interest has been developed in utilizing probiotics as colonizers to prevent the cycle of colonization with pathogens and ultimately nosocomial infections [7]. Thus the therapeutic concept with probiotics is an effort to reduce or eliminate potential pathogens and toxins, to release nutrients, antioxidants, growth factors and coagulation factors, to stimulate gut motility and to modulate innate and adaptive immune defense mechanisms via the normalization of altered gut flora [8].

Currently bacteria in synbiotic (prebiotic and probiotic components) and probiotic preparations are being used experimentally in the treatment of acute pancreatitis, liver transplantations, inflammatory bowel disease, acute and infectious diarrhea and trauma patients [9].

## Properties of Probiotics

Probiotics are, according to the FAO/WHO "live bacteria which when administered in adequate amounts confer a health benefit to the host". These bacteria do not contain any virulence properties or antibiotic resistance cassettes. They create an unfavourable environment for pathogens by mechanisms including promotion of the integrity of the guts defense barrier by normalizing intestinal permeability, modulation of intestinal secretory immunoglobulin function, control of intestinal inflammatory responses and by balancing the release of cytokines. In addition, probiotics maintain normal microecology of the gastrointestinal flora and antimicrobial effects mediated by nutrient competition, alteration of local pH, production of bacterocins, modification of pathogen derived toxins and stimulation of epithelial mucin production [7]. Numerous microorganisms (see Table 1 and Table 2; e.g. *Lactobacillus rhamnosus *GG, *L. reuteri*, bifidobacteria and certain strains of *L. casei *or the *L. acidophilus*-group) are used in probiotic food, particularly fermented milk products, or have been investigated--as well as *Escherichia coli *strain Nissle 1917, certain enterococci (*Enterococcus faecium *SF68) and the probiotic yeast *Saccharomyces boulardii*--with regard to their medicinal use. A well studied synbiotic is the preparation Synbiotic 2000 Forte. It contains the bacteria *Pedicoccus pentasacceus*, *Leoconostoc mesenteroides*, *Lactobacillus paracasei *subsp. *paracasei *and *Lactobacillus plantarum *(at a dose of 10^10 ^bacteria per sachet) as probiotics and betaglucan, inulin, pectin and resistant starch as prebiotics [5]. VSL#3 (VSL Pharmaceutical, Gaithersburg, Maryland) is a probiotic preparation containing live freeze- dried lactic acid bacteria containg approximately 450 billion bacteria in defined ratios of lyophilized *Bifidobacterium breve, B. longum, B. infantis, L. acidophilus, L. plantarum, L. casei, L. bulgaricus *and *Streptococcus thermophilus *Since probiotics are staying only transiently in the gut they have to be ingested every day. This is crucial as most antibiotic treatments will kill the probiotic bacteria [3].

**Table 1:** List of studies mentioned with no or negative effects of probiotics on critically ill patients

Study	Study design	Number of patients	Probiotics/Synbiotics	Results
Jain et al. [11]	double-blind, randomized, placebo-control.	90 ICU patients: 45 verum vs 45 placebo.	4 × 10^9 ^of *Lact. acidophilus, Bifid. lactis, L. bulgaricus, Strept. thermophilus +*7,5 g Oligofructose.	less potentially pathogenic bact. **no differences**: septic outcome mortality, ICU stay.

McNaught et al. [12]	nonblinded, randomized, nonplacebo.	103 critical lll: 51 verum vs 52 placebo.	*L. plantarum *299v + oatmeal + fruit drink.	**no differences**: intestinal permeability, CRP level morbidity + mortality.

Olah et al. [13]	double-blind, randomized, placebo-control.	62 patients with acute pancreatitis: 29 vs 33.	Synbiotic 2000 Forte.	**no differences**: mortality, septic complications, organ failure.

Barraud et al. [14]	double-blind, randomized, placebo-control.	167 ICU patients: 84 verum vs 83 placebo.	2 × 10^10 ^*Lact. rhamnosus *GG.	**no differences**: mortality, ICU acquired infections.

**Table 2:** List of studies mentioned with positive effects of probiotics on critically ill patients

Study	Study design	Number of patients	Probiotics/Synbiotics	Results
Alberda et al. [15]	double-blind, randomized, placebo-control.	28 ICU patients: 14 verum vs 14 placebo.	9 × 10^11 ^*L. casei, L. plantarum, Strept. salivarius, B. infantis L. acidophilus, B. longum, L. delbrueckii, B. breve*.	increase in IgA + IgG in verum group.

Spindler-Vessel et al. [16]	nonblinded, randomized, nonplacebo.	113 ICU trauma pat.: 87 verum vs 26 placebo.	10^10 ^of *P. pentosaceus*, 10^10 ^*L. paracasei*,10^10 ^*L. plantarum +*2,5 g ß-glucan, inulin, pectin, starch.	reduction in: pneumonias+ intestinal permeability + procalcitonin levels.

Olah et al. [17]	double-blind, randomized, placebo-control.	45 patients with acute pancreatitis: 23 vs 22	10^9 ^*L plantarum *299 vs heat killed bacteria *+*10 g oat fibre.	less septic complications in verum group.

Forestier et al. [18]	double-blind, randomized, placebo-control.	208 ICU patients: 104 verum vs104 placebo.	10^9 ^*Lact. rhamnosus*, 10^9 ^*Lact. casei*.	*P. aeruginosa *colonization + infection is delayed in verum.

Kotzampassi et al. [19]	double-blind, randomized, placebo-control.	65 ICU trauma pat.:32 verum vs 33 placebo.	Synbiotic 2000 Forte.	reduced rate of infection, SIRS, severe sepsis + mortality in verum.

Klarin et al. [20]	nonblinded, randomized, nonplacebo.	44 ICU patients: 22 verum vs 22	10^9 ^*Lact. plantarum *299v + oatmeal.	colonisation of *C. difficile *not detectable in verum group.

Morrow et al. [21]	double-blind, randomized, placebo-control.	146 ICU patients: 73 verum vs 73 placebo.	10^10 ^*Lact. rhamnosus *GG10^9 ^*Lact. casei*.	development of VAP less likely + less CDAD in verum group.

Frohmader et al. [22]	double-blind, randomized, placebo control.	45 ICU patients: 20 verum vs 25 placebo.	VSL#3.	effective in reducing frequency of liquid stool.

## Basic mechanisms of probiotics

During periods of critical illness, significant alterations occur in the gut microflora. Reasons for this are changes in circulating stress hormones, gut ischemia, the use of immunosuppressive drugs, antibiotics and the lack of adequate nutrients. Several studies have shown that certain probiotic bacteria are able to maintain the intestinal barrier function und to modulate the mucosal and intestinal immune function [3]. Probiotics may also reduce the incidence of VAP via a combination of local and systemic effects resulting in decreased colonisation [4]. The mechanisms are local and systemic. Local effects include reduced overgrowth of potential pathogens by competitive inhibition and direct antimicrobial effects. Secondary systemic benefits may result from improved gut mucosal barrier function, reduced bacterial translocation and upregulation of immune function [5,10,11]

Based on these properties of probiotics, several studies were conducted in the critical ill patients. We have conducted a PubMed search with the terms "Probiotic" and "Intensive care unit". We received 75 "hits" (end of Dec. 2010) and here we will present the most notable studies [Table 1 and Table 2].

### Evidence for no or negative effects on the outcome of critical ill patients

Jain and colleagues (2004) [11] demonstrated in a double-blind, randomized and placebo controlled study a "favourable" change in the colonization of gastric contents in 45 ICU patients treated with synbiotic preparation (4 × 10^9 ^of *Lactobacillus acidophilus, Bifidobacterium lactis, L. bulgaricus, Streptococcus thermophilus*) in comparison of 45 ICU patients receiving a placebo. Even though there was a significant difference in the number of aspirates with multiple organisms cultured (39% versus 75%) as well as the number of potentially pathogenic organisms (43% versus 75%) identified in the synbiotic group when compared to placebo, there were no significant differences between the two groups in the secondary outcomes of septic complications, mortality, days in ICU, days of hospitalization and respiratory complications.

No significant changes in cultures of the NGT aspirates and no significant differences with regard to intestinal permeability, CRP levels, septic morbidity and mortality or days in the ICU was reported in another study [12] with a preparation of *L. plantarum *299v (nonblinded, randomized, non placebo controlled study). However there was a significant difference in favour of the probiotic of IL-6 levels compared to control.

Olah and colleagues (2007) [13] used in their double-blind, randomized and placebo controlled study a high CFU synbiotic product Synbiotic 2000 Forte in 62 patients with severe acute pancreatitis. There were no statistical significant differences in the incidence of mortality, septic complications or development of multiorgan failure between the groups. However, the total incidence of SIRS and multiple organ failure was significantly different between the two groups (8 vs. 14). In addition, the rate of complications was significantly less in the verum group.

In a most recent study, Barraud and colleagues (2010) [14] conducted a double-blind, randomised and placebo-controlled trial to assess the effects of prophylactic probiotic administration in patients on mechanical ventilation. A total of 167 patients were included, half of these received 2 × 10^10 ^*Lactobacillus rhamnosus *GG. The 28 day mortality rates were not different in the probiotic (25,3%) and placebo groups (23,7%). The incidence of ICU-acquired infections did not differ significantly except for that of catheter related bloodstream infections that was lowered by probiotics.

### Evidence for positive effects on the outcome of critical ill patients

Alberda and colleagues (2007) [15] demonstrated a significant increase in IgG and IgA levels in favour of the live synbiotics (preparation of 9 × 10^11 ^*L. casei, L. plantarum, L. acidophilus, L. delbrueckii, Bifidobacterium longum, B. breve, B. infantis*, *Streptococcus salivarius*) over devitalized synbiotics or placebo in 28 ICU patients (double-blind, randomized, placebo controlled study).

Spindler-Vessel and colleagues (2007) [16] were able to demonstrate a significant reduction in infectious pneumonias (19% versus 53%), intestinal permeability, and procalcitonin levels in 113 trauma ICU patients with the use of a symbiotic preparation (10^10 ^of *P. pentosaceus*, 10^10 ^*L. paracasei*, 10^10 ^*L. plantarum*). This study was however nonblinded, randomized, nonplacebo controlled.

Olah and colleagues (2002) [17] performed a randomized double-blind study in 45 patients with acute pancreatitis. Significant results were demonstrated in favour of the live synbiotic (10^9 ^*L plantarum *299) for septic complications requiring surgery (5% versus 30%) and positive pancreatic aspiration cultures (5% versus 30%). However there were no significant differences in respiratory infections, rates of multiple organ failure, days of hospital stay, mortality, CRP levels, rates of necrotizing pancreatitis or positive blood cultures.

Forestier and colleagues (2008) [18] conducted a prospective, randomized, double-blind, placebo controlled pilot study with 208 patients on an ICU unit, with a stay longer than 48 hours. Through a nasogastric feeding tube patients received either 10^9 ^CFU *Lactobacillus casei/rhamnosus *or placebo twice daily. The authors showed that the occurrence of *P. aeruginosa *respiratory colonization and/or infection was significantly delayed in the probiotic group with differences in median delay to acquisition of 11 versus 50 days.

In another double-blind, placebo controlled trial the benefits of a Synbiotic 2000 Forte treatment was investigated on the rate of infections, SIRS, severe sepsis and mortality in critical ill patients. 65 patients were randomized to receive once daily for 15 days the synbiotic formulation. These patients exhibited a significant reduced rate of infections (63% versus 90%), SIRS, severe sepsis and mortality (14% versus 22%) in comparison to the placebo group [19].

The incidence of *Clostridium difficile*-associated diarrhoe (CDAD) in hospitalised and especially critical ill patients is increasing because of the wide use of antibiotics. In a Swedish study, 22 ICU patients were given the probiotic bacterium *L. plantarum *while 22 other patients received a placebo. 19% of the placebo group were colonised with *C. difficile*, while none were colonised who received the probiotic [20].

In a study which was published last year, Morrow and colleagues [21] elucidated if the use of *Lactobacillus rhamnosus *GG can reduce the incidence of VAP. They randomized 146 patients (prospective, double-blind and placebo-controlled study) and found that patients treated with the bacteria were significantly less likely to develop a VAP when compared to patients treated with a placebo. Also patients had significantly less CDAD than placebo treated patients (18,6% versus 5,8%) even though the duration of the diarrhoe was not different between the groups.

In a pilot trail with the probiotic mixture VSL#3 Frohmader and colleagues [22] described the effectiveness of these bacteria in reducing the frequency of liquid stool in critically ill patients receiving enteral nutrition. In this single center, double-blind, randomized and placebo controlled study the group included 45 adults (20 verum vs 25 placebo). The verum group had a significant reduction in the frequency of liquid stool (0.27 vs 0.93; P = 0.03).

## Conclusions

The use of probiotic or synbiotic preparations in critically ill patients continues to be controversial. However, we found fewer publications reporting no or negative effects on the use of probiotics in critical ill patients, than the number of publications presenting positive effects [Table 1 and Table 2].

The problem we are facing is that most of the current literature is limited by poor trial design, small study samples, poorly defined endpoints and a great variety of often poorly defined mixtures of probiotics. Additionally, the number of probiotic bacteria ingested by the patients vary up to a hundredfold from study to study. Studies were always from single centers and some of the results were rather weak; Olah et al [13] reported that the lower incidence of multiorgan failure, septic complications and mortality was not significant but the total incidence of SIRS and multiorganfailure was significantly different between the groups. Basically the studies included three different groups of patients; patients with acute pancreatitis, trauma patients and patients being admitted to the ICU with a whole range of severe medical problems (eg cardiac, respiratory). Because of the limited number of patients and the different probiotic preparations and the different endpoints (e.g. reducing rate of infection, SIRS, procalcitonin levels, development of VAP or CDAD), unfortunately, it is not possible to break down which group of patients will benefit most from the use of probiotics.

Well designed multi center studies with a defined mixture of bacteria, for example Synbiotic 2000 Forte, on a defined group of critically ill patients are essential before any conclusion on the effect of probiotics/synbiotics can be drawn. In addition, the endpoints have to be clearly defined. Clinical work is one step, but similarly important is the research on the bench to elucidate the exact mechanisms by which probiotic strains elicit their effects in the host organism. Once we understand these mechanisms then we might even go a step further to improve the properties by using "patho-biotechnology". Essentially, this novel approach involves the generation of "improved" probiotic strains, transforming them with stress survival systems from other microbes. A good example is cloning and heterologous expression of a single bile resistance gene from the food borne pathogen *Listeria monocytogenes *in the probiotic strain *Bifidobacterium breve*, not only improves gastrointestinal colonisation and persistence, but also significantly bolsters the clinical efficacy of the probiotic strain. In addition, 'designer probiotics' have been engineered to express receptor-mimic structures on their surface. When administered orally these probiotics bind to and neutralize toxins in the gut lumen and interfere with pathogen adherence to the intestinal epithelium - thus essentially preventing the infection. In this situation is the use of antibiotics unnecessary, circumventing the problems with antibiotic resistance [23,24].

In conclusion, we have not yet enough data to firmly support the use of probiotic bacteria in the setting of intensive care units. Well designed multi center clinical studies with defined mixtures of probiotics and defined endpoints are warranted in this field.

## Abbreviations

CDAD: *Clostridium difficile*-associated disease; CFU: colony forming unit; CRP: C-reactive protein; ICU: Intensive Care Unit; NGT: Nasogastric tube; SIRS: Systemic inflammatory response syndrome; VAP: Ventilator associated pneumonia

## Competing interests

The authors declare that they have no competing interests.

## Authors' contributions

CAJ surveyed literature and developed text draft of the review. CS refined the write up, and PM supervised the whole process. All authors have read and approved the final manuscript.
